# Genomic Diversity and Evolution of Identified SARS-CoV-2 Variants in Iraq

**DOI:** 10.3390/pathogens13121051

**Published:** 2024-11-29

**Authors:** Ahmed A. Al-Mankhee, Yassmin Moatasim, Ahmed El Taweel, Mokhtar Gomaa, Omar A. Rabiee, Marwa M. Gado, Ahmed B. Barakat, Mohamed A. Ali, Rabeh El-Shesheny

**Affiliations:** 1 Al Hussein Teaching Hospital, Al Bat’haa 64011, Thi Qar, Iraq; ahmedabdulrazzaqabbas@gmail.com; 2 Department of Microbiology, Faculty of Science, Ain Shams University, Cairo 11566, Egypt; omaralfarouk@sci.asu.edu.eg (O.A.R.); marwagado@sci.asu.edu.eg (M.M.G.); dr.barakat51@hotmail.com (A.B.B.); 3 Center of Scientific Excellence for Influenza Viruses, National Research Centre, Giza 12622, Egypt; yasmin.moatasim@human-link.org (Y.M.); ahmed.nageh@human-link.org (A.E.T.); mokhtar.rizk@human-link.org (M.G.)

**Keywords:** SARS-CoV-2, genomic, Iraq

## Abstract

The COVID-19 pandemic caused by the SARS-CoV-2 virus continues to circulate worldwide, causing the deaths of millions of people. The continuous circulation of the virus, its genetic diversity, the emergence of new variants with increased transmissibility, and/or the capacity of the virus to escape from the immune system constitute a major public health concern. In our study, we aimed to characterize SARS-CoV-2 strains in Iraq from the first introduction until the end of 2023, and to identify their variants, lineages, clades, and mutation patterns. All published Iraqi full genome sequences (2020–2023) were obtained from Global Initiative on Sharing All Influenza Data (GISAID) and subjected to molecular characterization along with 19 samples of full genome sequences that were collected during the fifth and sixth waves of the SARS-CoV-2 pandemic in this study. Next-generation sequencing was performed using an Illumina MiSeq system, and phylogenetic analysis was performed for all the Iraqi sequences. Three established global platforms, GISAID, Nextstrain, and PANGO, were used for the classification of isolates into distinct clades, variants, and lineages. Six wave peaks of COVID-19 cases have been identified in Iraq, resulting in approximately 2,400,000 cumulative confirmed cases and more than 25,000 deaths. Our study revealed patterns of circulation and dominance of SARS-CoV-2 clades and their lineages in the pandemic waves in the country.

## 1. Introduction

In December 2019, the first cases of severe acute respiratory syndrome coronavirus 2 (SARS-CoV-2) were reported in Wuhan, China. The SARS-CoV-2 pandemic has significantly affected the world, disrupting health systems, economies, businesses, science, tourism, and other aspects of life [1].

SARS-CoV-2 is a positive single-stranded RNA virus that belongs to the β-genus of the Coronaviridae family. RNA viruses are known for their high genetic mutation rate, allowing them to evade host immunity, which can complicate the development of an effective vaccine and the development of fast and stable reverse transcription-quantitative PCR (RT-PCR) techniques for coronavirus disease 2019 (COVID-19) detection [2,3]. RNA viruses pose a significant public health threat due to their high transmission rates, high mutation rates, and aggressive competition with host cellular functions. As a result of SARS-CoV-2 mutation dynamics globally, several variants of concern (VOC) have emerged, showing evidence of enhanced viral transmission. These variants have been linked to increased rate of hospitalization, more frequent admissions to critical care units, and varying degrees of mortality compared with the wild-type Wuhan-1 strain [4,5,6].

Several variants of SARS-CoV-2 have been reported globally, resulting from one or more mutations in the viral genome that can affect the viral transmissibility, virulence, and the host’s immune response [7]. A SARS-CoV-2 variant of proven or suspected clinical or epidemiological relevance is designated as a variant of interest (VOI) based on criteria such as increased transmissibility and ability to escape immunity. Increased transmissibility is of particular concern, as it leads to higher infection rates and may necessitate the implementation of more stringent public health measures [8]. Variant escape from antibody neutralization can compromise the effectiveness of vaccination programs, requiring the development of modified vaccines or the use of booster doses. Variants with a combination of these characteristics have a considerable impact on pandemic management [9,10].

Five VOCs have circulated globally and have become the predominant variants in the regions where they were initially identified [11]. The alpha variant (also known as B.1.1.7, VOC202012/01, or GRY) was first detected in the United Kingdom in September 2020 and has since been reported in 179 countries. The beta variant (also known as B.1.351 or GH/501Y.V2) [12] was first reported in South Africa in October 2020 and has since been reported in 120 countries. The gamma variant (also known as P.1 or GR/501Y.V3 (B.1.1.28) was first reported in Brazil and has since been reported in 92 countries. The Delta variant (also known as B.1.617.2 or G/478K.V1) was first reported in India and has since been identified in 188 additional countries. The omicron variant (also known as B.1.1.529 or GRA) was detected in November 2021 in 119 countries. Each variant has spread across multiple continents, with omicron currently being the most prevalent VOC in many countries, including the United Kingdom, the United States, and many European countries [12,13,14,15,16]. The rapid spread of alpha, delta, and omicron in particular strongly suggests that these variants have transmission advantages over the ancestral viruses. Based on modeling data, the alpha variant is estimated to be 43–90% more transmissible than previously circulating variants, while the delta variant is believed to be around 60% more transmissible than alpha [17,18].

The SARS-CoV-2 omicron VOC is highly transmissible, with early estimates suggesting that it could be around 100% more transmissible than the delta variant. Preliminary studies indicate that omicron may be associated with a lower risk of hospitalization and reduced disease severity compared to delta, although it remains unclear how much of this is due to increasing population immunity over time. Nevertheless, omicron should not be regarded as only causing mild disease as its rapid spread can overwhelm healthcare systems [19,20,21].

In Iraq, the first confirmed case of COVID-19 was reported on February 24th 2020 in Al-Najaf city in the south of Baghdad [22]. From 3 January 2020 to 1 October 2023, more than 2.4 million confirmed cases and more than 25,000 deaths due to SARS-CoV-2 were reported to the WHO [23]. Therefore, identifying the SARS-CoV-2 genome sequences and understanding their mutations are important. However, only a limited number of SARS-CoV-2 sequences have been retrieved from Iraq and included in the international database (GISAID, https://gisaid.org/ (accessed on 27 November 2024)). This study aimed at investigating the molecular epidemiology of SARS-CoV-2 variants and the evolution of variants circulating in Iraq between June 2020 and November 2023. Sequencing of SARS-CoV-2 samples from the Thi-Qar Governorate of Iraq from the fifth and sixth waves of the pandemic, which peaked in the summer period of 2022 in the country, was performed for the identification of dominant variants, clades, and lineages and to reveal the potential mutation patterns in the sequences of SARS-CoV-2.

## 2. Materials and Methods

### 2.1. Sample Collection

Samples were collected from patients suspected of having COVID-19 who were symptomatic or had known exposure to the disease using nasopharyngeal (NP) swabs. Between August 2022 and March 2023, laboratory technicians in the Thi-Qar Governorate, southern Iraq, collected 90 clinical samples (56 males and 34 females, between 18 and 64 years old) from outpatients, inpatients, and emergency departments at Al Hussein Teaching Hospital and the Central Public Health Laboratory, affiliated to the Thi-Qar Health Department. All samples were transferred to a clinical laboratory for SARS-CoV-2 testing. Collected samples were processed and subjected to genetic material extraction using an Applied Biosystem Mag max viral detection kit and KingFisher^®^ Flex extraction machine (Thermo Fisher Scientific, Rocklin, CA, USA). SARS-CoV-2 was detected using a Real-time RT-PCR detection kit using N gene and ORF1ab primers and probes (Certest Biotec SL, Zaragoza, Spain). Positive samples that had a high copy number of the virus with cycle threshold (Ct) values < 25 were selected for sequencing.

### 2.2. SARS-CoV-2 Whole Genome Sequencing

First, cDNA strands of the viral genome of each sample were synthesized and double strands were amplified using SuperScript™ IV One-Step RT-PCR System (Invitrogen, Waltham, MA, USA). After PCR purification and clean-up, we used the Illumina Nextera XT DNA library prep kit for sequencing on the MiniSeq Illumina Sequencing System (Illumina, San Diego, CA, USA). No sample included a mixed population of viruses, and every sample generated enough strain-typeable sequences. CLC Genomics Workbench version 20 (CLC Bio, Qiagen, Aarhus, Denmark) through workflow was then used to align the reads with the reference genome (NC_045512.2). Next generation sequencing (NGS) was performed at the Center of Scientific Excellence for Influenza Viruses (CSEIV) laboratory at the National Research Centre, Egypt, and whole genome sequences were submitted to the GISAID platform under Accession numbers EPI_ISL_18858302-EPI_ISL_18858315 and EPI_ISL_18861671-EPI_ISL_18861675.

### 2.3. Sequence Alignment and Phylogenetic Analysis

Full genome sequences and metadata of Iraqi SARS-CoV-2 viruses are available on the GISAID Initiative (EpiCoVTM) website, as only some are shown in Table 1. On, 10 August 2023, the complete genomes of SARS-CoV-2 viruses collected from various governorates from Iraqi patients were retrieved from the GISAID initiative database (EpiCoVTM). A total of 1041 virus genomes were retrieved https://clades.nextstrain.org/ (accessed on 10 August 2023) [24]. The GISAID platform provides high-coverage sequences of Iraqi SARS-CoV-2 genomes, which were used to estimate the phylogeny of full genomes. The alignment of reference sequences and consensus sequences from the whole genomes of SARS-CoV-2 strains was conducted using the MAFFT web server (https://mafft.cbrc.jp/alignment/server, accessed on 3 January 2024). A sequence alignment file and IQ-TREE 1.6.1 were used to build an inference maximum-likelihood tree. The phylogenetic tree was built using the FigTree v1.4.2 program (http://tree.bio.ed.ac.uk/software/Figtree, accessed on 3 January 2024) to enhance its appearance.

### 2.4. Sequence Data Lineage Classification

The entire set of Iraqi genomes, together with their metadata, were retrieved from the GISAID website. The genomes were collected between 5 June 2020 and 1 September 2023. The lineage and sub-lineage of the samples that were collected were classified using Pangolin (https://cov-lineages.org/, accessed on 3 January 2023) and NextStrain (https://nextstrain.org/blog/2021-01-06-updated-SARS-CoV-2-clade-naming (accessed on 3 January 2023)) criteria. The complete Iraq sequences database from June 2020 to February 2024 and/or the GISAID database (https://www.gisaid.org/, accessed on 3 January 2023) were used to look up novel mutation combinations for viruses that were similar.

## 3. Results

### 3.1. Epidemiology of SARS-CoV-2 in Iraq

**Figure 1:**
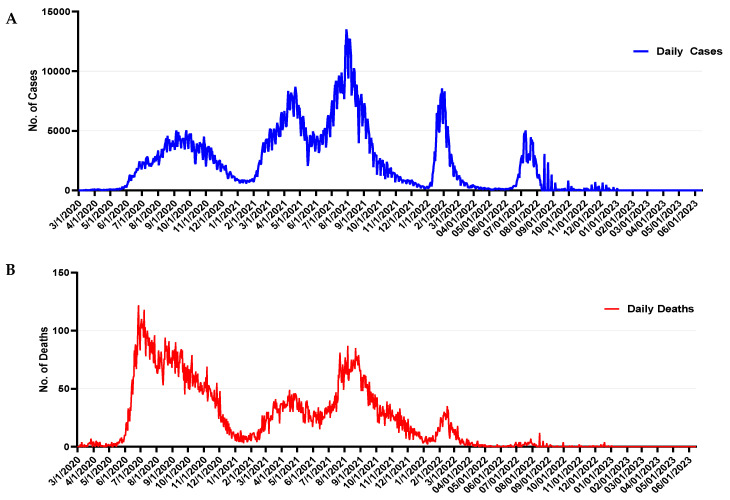
Daily COVID-19 confirmed cases (**A**) and deaths (**B**) in Iraq from March 2020 to July 2022. Data extracted from the WHO.

Based on the available epidemiological data, six waves of SARS-CoV-2 infection were detected in the Iraqi population. The number of confirmed infections in Iraq, according to the WHO, reached approximately 2.4 million cases, with more than 25,000 deaths (Figure 1). The waves of the pandemic were subsequently distributed. The pandemic started in April 2020 and the country witnessed the beginning of the first wave, which reached its peak by August 2020. Those infected experienced severe symptoms, and the death rate was significant during this wave. The second wave coincided with the onset of winter weather in early 2021. The third reported wave peaked in August 2021, and this wave had a higher case fatality rate compared to the second wave. The subsequent waves in 2022 were also linked but they were less severe in terms of symptom severity and mortality (Figure 1).

### 3.2. Timeline Distribution of SARS-CoV-2 Genome Sequences in Different Iraqi Governorates

**Figure 2:**
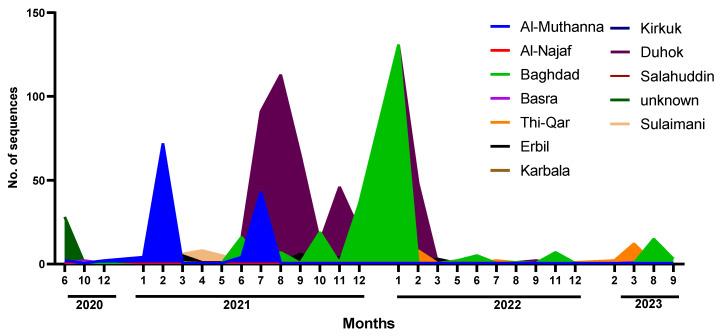
Timeline distribution of SARS-CoV-2 genome sequences in different Iraqi governorates (March 2020 to September 2022).

The distribution of the 1041 genome sequences that were retrieved from the GISAID platform were spread among multiple geographical areas in northern, southern, and central Iraq; the central governorates of Iraq had 252 records, the majority of which were reported from Baghdad Governorate (244); 604 cases were from the north, with Dohuk Governorate having the highest proportion (540 cases). There were 185 cases from the southern governorates, most of which were from Al-Muthanna Governorate (157 cases) and Thi Qar Governorate (27 cases) (Figure 2).

### 3.3. Clades and Lineage Distribution of SARS-CoV-2 in Iraqi SARS-CoV-2 Sequences

**Figure 3:**
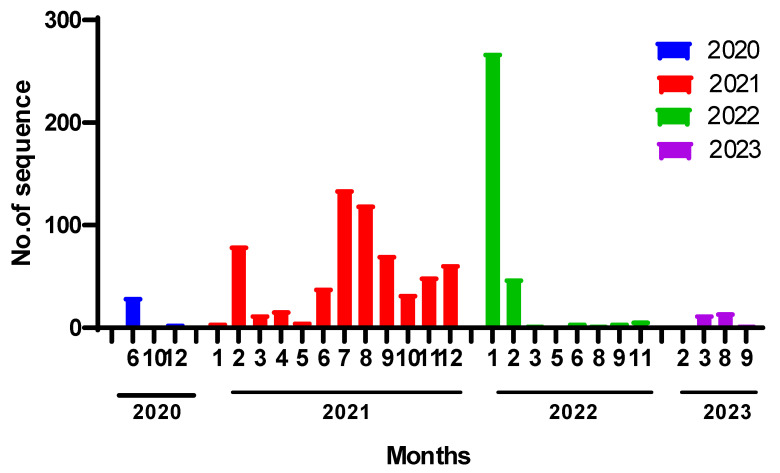
Timeline distribution of SARS-CoV-2 collective number of genome sequences in Iraq (March 2020 to September 2022).

Along with our 19 sequenced isolates in this study, the published 1022 whole genome sequences of SARS-CoV-2 strains from Iraq were downloaded from GISAID until December 2023 and then subjected to genetic analysis using Nextclade (v2.14.1; https://clades.nextstrain.org/, accessed on 27 November 2024) and using Wuhan-Hu-1/2019 strain as a reference strain. Fully sequenced Iraqi genomes yearly distribution in the database was as follows: 36 (2020), 631 (2021), 344 (2022), and 30 (2023) sequences (Figure 3).

According to GISAID clade analysis, all Iraqi SARS-CoV-2 sequences were depicted genetically in nine clades: GK (n = 519), GRA (n = 328), GRY (n = 96), GR (n = 46), GH (n = 43), G (n = 3), O (n = 3), GV (n = 2), and L (n = 1) (Figure S1). In 2020, four clades circulated, with the GH clade being the most prevalent. During 2021, around eight clades circulated, with GRY (Alpha) and GK (delta) being the most prevalent. In 2022, there were four branches, with GRA (omicron variant) being the most prevalent and continuing to circulate during 2023.

**Figure 4:**
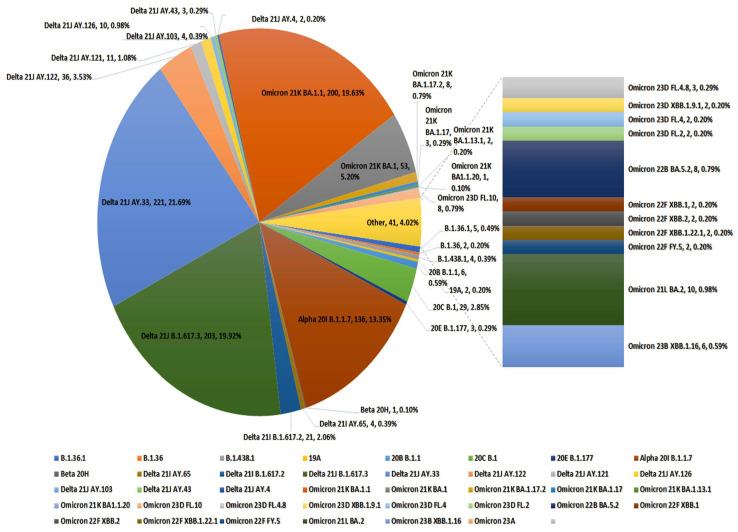
Distribution of all full genome SARS-CoV-2 variants in Iraq. Represented as WHO clades, followed by Next clade system and PANGO system, number of sequences and present of presence in the 1041 sequences.

According to WHO clades classification, 49.76% of the sequences are delta, (32%) are omicron, while 136 sequences (13%) are alpha. 20C represented 3% (29 sequences), 20A represented 2% (16 sequences), and 20B represented 1% (8 sequences). Beta, 19A, and 20E were represented by only 6 sequences (Figure 4). Moreover, 61 lineages have been identified in the genomes investigated by applying the Pangolin COVID-19 platform. The most prevalent lineages were determined to be AY.33 (n = 227), B.1.617.2 (n = 217), BA.1.1 (n = 214), B.1.1.7 (n = 132), AY.122 (n = 36), BA.1 (n = 36), B.1.428.1 (n = 29), AY121 (n = 11), BA.2 (n = 10), B.1.1 (n = 9), BA.5.2 (n = 8), BA.1.17.2 (n = 8), FL.10 (n = 8), B.1 (n = 7), XBB.1.16 (n = 5), and FL4.8 (n = 3), XBB.122.1 (n = 2), FY.5 (n = 2), B.1.351 (n = 1), and BA.1.20 (n = 1). The distribution of all Iraqi published sequences lineages and clades are shown in Figure 4.

### 3.4. SARS-CoV-2 Lineages in Epidemic Waves Among the Iraqi Population

All published and laboratory sequence lineages and clades were classified throughout the time range of the detected waves (Figure S2). The first wave was mainly represented by 20C clade, B.1.428.1 lineage (71%), followed by 20A (10%), 20 I-alpha (12%), 20E (5%), and 19A (2%). For the second wave, 20 I-alpha was the most represented (78%), mainly through B.1.1.7 lineage (75%), followed by clade 20A (8%), 20 B (7%), 21J-delta (5%), and 19A and 20E (1%). Wave 3 was mainly represented by 21J-delta sequences (87.1%), followed by 20I-alpha (7.7%), 21I-delta (4.1%), and 21K-omicron sequences (1%). Lineages AY.33 and B.1.617.2 represented 42% and 38% of wave 3 sequences, respectively. Wave 4 sequences are mainly 21K-omicron (84.1%) of which 66% belong to BA.1.1 lineage, followed by clade 21J-delta (13.2%), 21L-omicron (3.5%), and 21I-delta (1.3%) of all wave 4 sequences. Wave 5 sequences are mainly 22B-omicron (66.7%) and specifically lineage BA.S.2 (47%), followed by 21L-omicron (13.3%), and 22D-omicron (6.7%), with low representation of 21J-delta and 20A (6.7% each). Wave 6 only was represented by omicron sequences, 23D (47.5%), 22F (27.5%), 23B (15%), 23A (5%), and 22B (2.5%) of wave 6 sequences.

### 3.5. Genomic Variation and Mutation Signature in Iraqi SARS-CoV-2 Sequences

Our analysis revealed that the D614G spike mutation had the highest frequency and was present in all Iraqi sequences. This mutation improved spike protein fitness to cell surface receptors and increased the virus’s transduction compared to the wild type. Other predominant spike protein sequence variations are listed in Table 1. Other structural and non-structural protein sequences also showed high level of variations over time, as listed in Table S2.

### 3.6. Phylogenetic Analysis of SARS-CoV-2 from Iraq

**Figure 5:**
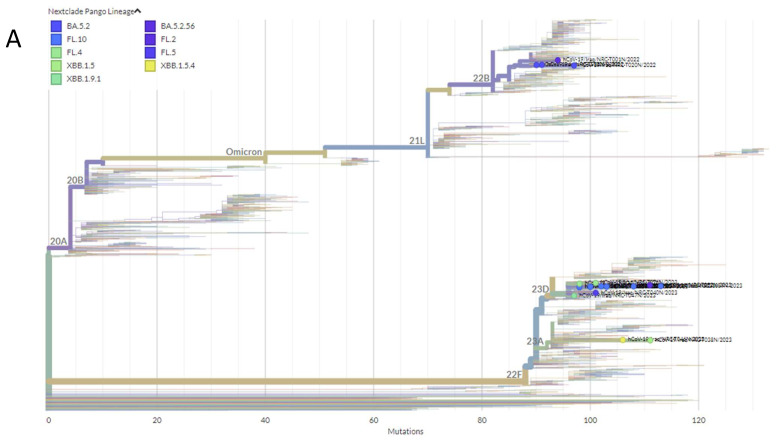

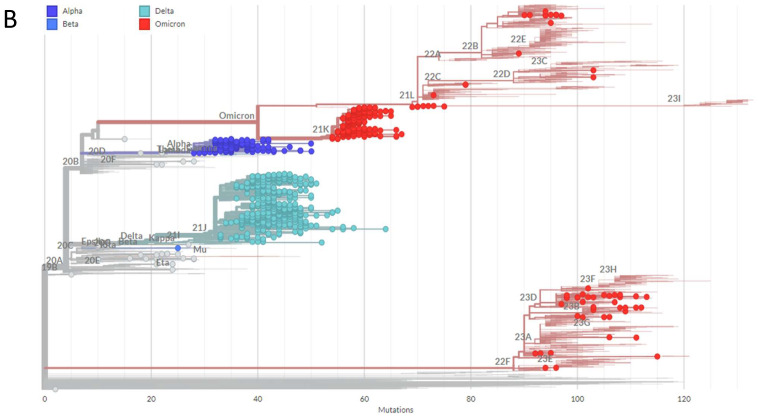
Phylogenetic SARS-CoV-2 variant analysis. The phylogenetic tree was constricted based on sequence date in GISAID data using the Nextstrain server (Nextclade tool). Phylogenetic analysis tree for sequences obtained in this study (**A**) Phylogenetic analysis tree for all Iraqi published sequences (**B**).

According to the Nextclade analysis of SARS-CoV-2 sequences based on variants of concern from 1041 whole genome sequences from Iraq, the phylogenetic analysis tree showed that delta (B.1.627.2) and omicron (BA.5) are the most common variants (518 and 328, respectively); alpha (B.1.1.7) was represented in 136 sequences but the beta variant (B.1.351) had only one genetic sequence for all Iraqi published sequences (Figure 5A). The 19 genome sequences obtained from this study belonged to the omicron strain, and were categorized into three clades: the first clade, 23D, has 13 sequences, the second clade, 22B, has 4 sequences, while the third clade, 23A, has only 2 sequences. The 19 obtained sequences are divided into nine lineages according to Pang lineage sequences and were representative as follows: FL.10, BA.5.2, FL.4, FL.2, FL.5, BA.5.2.56, XBB.1.5, XBB.1.5.4, and XBB.1.9.1, as shown in Figure 5B.

## 4. Discussion

Genomic surveillance of SARS-CoV-2 plays a critical role in understanding and responding to the pandemic. Tracking the emergence of mutations and variants through whole genome sequencing enables early detection of novel variants of concern and monitoring their spread, allowing public health officials to implement timely tailored containment measures.

The current study investigated the molecular epidemiology of SARS-CoV-2 variants and the evolution of variants circulating in Iraq between June 2020 and November 2023. We characterized the genomic variation as well as the phylogenetic analysis of SARS-CoV-2 viruses that are circulating and causing pandemics worldwide. Due to the widespread transmission of and the multiple mutations in the SARS-CoV-2 genome, numerous SARS-CoV-2 variants have been detected, contributing to a higher risk of infection [1]. Several variants of the virus have been recorded around the world since the first identification of the virus in December 2019 [2,3]. Complete genome sequencing and phylogenetic analysis of SARS-CoV-2 strains is essential for tracking the virus evolution and understanding the circulation of SARS-CoV-2 variants. According to GISAID, there is little genetic information about the SARS-CoV-2 outbreak in Iraq. Therefore, the current study showed molecular characterization and mutation patterns of the circulating SARS-CoV-2 strains in all governorates of Iraq throughout all waves of the coronavirus pandemic when the daily cases reached their highest peak according to WHO. Also, this study examined the genome sequences of isolates and changes in the SARS-CoV-2 genome that were considered necessary for the monitoring, control, and treatment of SARS-CoV-2 infection. High rates of genome mutation are found in RNA viruses, which are frequently used to adapt to changing environmental conditions [4], evade the host’s immune response, and counter the effects of antiviral drugs [5].

In June 2020, the first SARS-CoV-2 genome sequence was reported during the first wave in Iraq, and belonged to the B.1/GH clade from the global epidemic, while B.1.428.1 appeared to be a prevalent lineage in the second half of 2020, which is designated as an Iraqi lineage and is a subtype of B.1.428 (known as Danish lineage according to the Pango lineages database: https://cov-lineages.org/ (accessed on 10 August 2023)) [6]. This lineage was first reported in April 2020 in Qatar [7]. The genomic diversity of the B.1.428.1 lineage was characterized by possessing several mutations in the genome, including two mutations in the spike protein region, which are D614G and A544N. D614G was the first mutation in the spike glycoprotein and was first identified in Germany in January 2020 and became the dominant mutation in all the circulating strains worldwide by June 2020 [8]. The D614G mutation can increase the transmissibility and infectivity of the virus compared to the wild type [9,10]. This mutation in the spike protein might affect the correct diagnosis of the virus infection, and also the severity of the disease [11]. In the first wave, other variants such as B.1.36, and its sub lineage B.1.36.1, appeared in early 2021. The B.1.36 lineage was first reported in Saudi Arabia in February 2020 [12]. The second wave was represented by the Alpha variant or appeared to be dominant mainly by B.1.1.7, accounting for about 76.25% of the SARS-CoV-2 complete genome sequences submitted to the GISAID platform during the first half of the year 2021. However, the lineage B.1.428.1, which appeared to be dominant in 2020, was not detected among the sequenced samples from 2021. Considering that B.1.1.7 has an increased transmissibility and pathogenicity, this could explain the reason for the increase in COVID-19 cases in early 2021 in Iraq compared to 2020 [13].

Subsequently, the delta variant of concern started to spread over the country and dominated the circulating variants of all the 1041 sequenced samples; 518 (49%) isolates were found to carry the delta variant. The 21J clade, in general, was dominant in the third wave, especially at the beginning of the summer of 2021, when the country was going through the worst wave of the pandemic, compared to the SARS-CoV-2 isolates in early 2021. During this wave, B.1.617.2 and AY.33 of the delta variant were the predominant circulating lineages based on the sequences from Iraq. This variant had been detected before in the Thi-Qar and Erbil Governorates at the end of April and June 2021, respectively. It was determined that the delta variants, which initially emerged in India in late 2020, possessed greater transmissibility and infectivity [14]. T478K mutation is shared in both delta (B.1.617.2) and omicron (B.1.1.529) variants present in the S gene of the virus but not in alpha (B.1.1.7). It has been demonstrated that this mutation improves ACE2 binding and transmissibility. Moreover, it facilitates the virus’s escape from the host’s defense mechanism [9]. Elevated COVID-19 cases in Iraq during this period may be caused by reducing lockdowns and encouraging individuals to go back to regular social life actions, which include handshakes, visiting funerals, and attending wedding ceremonies [15].

Our 19 whole genome sequences in this study were taken from the fifth and sixth waves of the pandemic in the country. According to the Nextclade platform, all 19 sequences belonged to the Omicron strain, as it was revealed through comparison with the first Omicron strain BA.1, belonging to clade 21K, that appeared early in Dohuk Governorate, northern Iraq, in late November 2021, which coincided with the first appearance of Omicron globally and that contains 55 mutations in the spike protein and other proteins and about 100 mutations in the entire genome, which led to these VOCs being more transmissible and causing immune escape. According to Pang lineage sequences, omicron variantFL10 from the sixth pandemic wave was clustered as 23D (Omicron variant), which had not been identified in the country before and was detected in eight sequences during February and March 2023; after that, four isolates of BA.5.2 were diagnosed in the fifth wave, which belong to the 22B clade of the Omicron strain, which was discovered in the country for the first time during the summer of 2022 in Baghdad Governorate. According to the GISAID platform, we report the first confirmed case of the FL.4 lineage that belonged to the 23D clade from omicron variant in Iraq, in a sample collected on 21 March 2023. The other isolates, represented by FL.2 and FL.5, which also belonged to the 23D clade, were diagnosed for the first time in Iraq during March 2023 in our isolates. Finally, XBB.1 isolate, which belongs to clade 23A, appeared for the first time in the country at the end of 2022 in Baghdad Governorate.

Our study revealed some mutations that were not present in the omicron variant in previous waves and are among the most important mutations in the spike protein: Q954H, T376A, and R408S substitutions were represented in 100%, and V83A, Q183E, F490S, N460K, and F486P substitutions, present in 78% of all 19 Thi-Qar sequences, while R346T and V213G mutations were present in 72% and 22%, respectively. Our results are in line with recent findings suggesting that the Q954H change in HR1 reduces rather than enhances fusion efficiency [25,26]. The N and M genes did not change compared to the first appearance of the Omicron BA.1 strain in Iraq, while in the E gene, a 80% replacement occurred at the T11A substitutions.

Overall, it was noted that the isolates from 2022 showed a rise in genetic diversity and a variety of viral lineages. Such an increase in viral genome diversity is derived from the mutations that occur due to viral replication [21]. Finally, this study had limitations, such as a small sample size and the impossibility of whole genome sequencing for every sample. Therefore, 19 samples were selected, which were the samples that covered the most complete genomes. These results could be important for epidemiological and molecular studies and for vaccine development and diagnostic approaches and could be shared in the global database for further studies and surveillance purposes. Furthermore, sequencing and monitoring of the SARS-CoV-2 virus and many other pathogens in Iraq is very slow due to the lack of expertise in sequencing facilities and funding. Research should focus on the impact and function of such genomic variants on virus infectivity, pathogenesis, and severity. Nevertheless, new vaccines, especially multivalent vaccines containing multiple VOCs, could be a good step forward in controlling the latest infections.

The detection of the omicron BA.2 subvariant in Iraq during the fifth wave of COVID-19 in January 2022 marks a significant and unique advancement. This finding highlights the importance of continuous genomic monitoring efforts, international data sharing, and cataloging of emerging variants and subvariants. These steps are crucial for promoting prompt public health responses and mitigating the impact of the pandemic.

## Figures and Tables

**Table 1:** Genetic variations in spike protein of SARS-CoV-2 in all Iraqi sequences as compared to Wuhan-1 strain.

Spike		
OMICRON	DELTA	OTHER
D614G (100%), P681H/R (94%), and T478K/R (79%)		
**All Omicron** G339D/H, S371L/F, S375F, K417N, N440K, S477N, T478K/R, E484A, Q498R, N501Y, Y505H, H655Y, N679K, P681H, N764K, D796Y, Q954H, and N969K (>98%)	**All Delta** E156- F157- Deletion, and L452R (100%) T19R, E158G, P681R (99%), G142D (90%), T478K (97%), D950N (84%)	**Alpha** N501Y (82%), A570D (98%), D614G (99%), P681H (94%), T716I (93%), S982A (97%), D1118H (94%), deletion of H69- V70- (94%), Y144- (97%) G181A (3%) **20C** A544N (100%) **20A** Q675H (75% of B1.438.1)
**21K** T95I, Y145D, L212I, G446S, Q493R, G496S, TS47K, and N856K (100%) Deletions of (H96- V70-, G142- V143- Y144-, N211-) (100%) Insertion of (EPE) at site 214 (100%). **21L** T19I, A27S, V213G, T376A, Q493R (100%) Deletion of L24- P25- P26- (100%), G142D (80%), D405N (83%), R408S (90%) **2B** deletion of H96- V70- (100%) L452R, F486V (100%) **22D** Deletion of L24- P25- P26- (100%) A27S, R346T, T376A, D405N, R408S (100%) **22F, 23B, and 23D** V83A, DEL Y144-, H146Q, Q183E, V213E, G252V, L368I, T376A, D405N, R408S, V445P G446S N460K, F486P, and F490S (100%) Deletion of L24- P25- P26- (94%) R346T (95%) P521Q (83% of 23B)	**21J** T95I (100% of AY.121 and AY.126) I850L (100% of AY.126) T29A and T250I (100% AY.33) L5F (6% of B.1.617.2), T29A (5% of B.1.617.2), D80Y (71% of B.1.617.2), T95I (87% of B.1.617.2), V1104L (11% of B.1.617.2) **21I** A222V and V1264L (100% AY.65) A222V 90% B.1.617.2 S494L 95% B.1.617.2 P600A 20% B.1.617.2	

## Data Availability

All data used in this study are available on request from the corresponding author.

## Supplementary Materials

The following supporting information can be downloaded at: https://www.mdpi.com/article/10.3390/pathogens13121051/s1, Table S1: The accession numbers of the viral genome, collection date, Pangoline lineage, World Health Organization (WHO) name, and Nextstrain clade of sequenced samples in this study. Table S2: Genetic variations in Other structural and non-structural of SARS-CoV-2 in all Iraqi sequences of SARS-CoV-2 as compared to Wuhan-1 strain. Figure S1: Distribution of SARS-CoV-2 variants in Iraq during six pandemic waves assigned using the GISAID clades, Next clade system, and PANGO system. Figure S2: Distribution of SARS-CoV-2 sequence lineages and clades throughout the different waves of infection in Iraq.
