# Supplementary material for: Genomic Diversity and Evolution of Identified SARS-CoV-2 Variants in Iraq
Source: Pathogens. 2024 Nov 29;13(12):1051. doi: 10.3390/pathogens13121051 (PMC11728743; doi:10.3390/pathogens13121051)
Supplement: Supplementary file 1 [file pathogens-13-01051-s001.zip › pathogens-3308383 supplementary/Figure S2.docx]

 **Figure S2.** Distribution of SARS-CoV-2 sequence lineages and clades throughout the different waves of infection in Iraq.
