# Supplementary material for: Genomic Diversity and Evolution of Identified SARS-CoV-2 Variants in Iraq
Source: Pathogens. 2024 Nov 29;13(12):1051. doi: 10.3390/pathogens13121051 (PMC11728743; doi:10.3390/pathogens13121051)
Supplement: Supplementary file 1 [file pathogens-13-01051-s001.zip › pathogens-3308383 supplementary/Table S1.docx]

**Table S1.** The accession numbers of the viral genome, collection date, Pangoline lineage, World Health Organization (WHO) name, and Nextstrain clade of sequenced samples in this study.

| **Virus name** | **Accession numbers** | **Pangoline lineage** | **Nextstrain clade** | **WHO name** | **Collection date** |
| --- | --- | --- | --- | --- | --- |
| hCoV-19/Iraq/NRC-T037N/2023 | EPI_ISL_18858302 | FL.10 | 23D | Omicron | 2023-02-20 |
| hCoV-19/Iraq/NRC-T038N/2023 | EPI_ISL_18861671 | XBB.1.5 | 23A | Omicron | 2023-03-12 |
| hCoV-19/Iraq/NRC-T040N/2023 | EPI_ISL_18858303 | FL.5 | 23D | Omicron | 2023-03-13 |
| hCoV-19/Iraq/NRC-T043N/2023 | EPI_ISL_18858304 | FL.10 | 23D | Omicron | 2023-03-14 |
| hCoV-19/Iraq/NRC-T046N/2023 | EPI_ISL_18858305 | XBB.1.9.1 | 23A | Omicron | 2023-03-16 |
| hCoV-19/Iraq/NRC-T047N/2023 | EPI_ISL_18858306 | XBB.1.5.4 | 23D | Omicron | 2023-03-16 |
| hCoV-19/Iraq/NRC-T052N/2023 | EPI_ISL_18861672 | FL.2 | 23D | Omicron | 2023-03-18 |
| hCoV-19/Iraq/NRC-T057N/2023 | EPI_ISL_18858307 | FL.10 | 23D | Omicron | 2023-03-19 |
| hCoV-19/Iraq/NRC-T058N/2023 | EPI_ISL_18858308 | FL.10 | 23D | Omicron | 2023-03-20 |
| hCoV-19/Iraq/NRC-T060N/2023 | EPI_ISL_18861673 | FL.4 | 23D | Omicron | 2023-03-20 |
| hCoV-19/Iraq/NRC-T063N/2023 | EPI_ISL_18858309 | FL.10 | 23D | Omicron | 2023-03-21 |
| hCoV-19/Iraq/NRC-T068N/2023 | EPI_ISL_18858310 | FL.10 | 23D | Omicron | 2023-03-22 |
| hCoV-19/Iraq/NRC-T076N/2023 | EPI_ISL_18858311 | FL.4 | 23D | Omicron | 2023-03-23 |
| hCoV-19/Iraq/NRC-T080N/2023 | EPI_ISL_18858312 | FL.10 | 23D | Omicron | 2023-03-23 |
| hCoV-19/Iraq/NRC-T089N/2023 | EPI_ISL_18861674 | FL.10 | 23D | Omicron | 2023-03-25 |
| hCoV-19/Iraq/NRC-T001N/2022 | EPI_ISL_18861675 | BA.5.2 | 22B | Omicron | 2022-08-02 |
| hCoV-19/Iraq/NRC-T020N/2022 | EPI_ISL_18858313 | BA.5.2.56 | 22B | Omicron | 2022-09-04 |
| hCoV-19/Iraq/NRC-T025N/2022 | EPI_ISL_18858314 | BA.5.2 | 22B | Omicron | 2022-09-19 |
| hCoV-19/Iraq/NRC-T036N/2022 | EPI_ISL_18858315 | BA.5.2 | 22B | Omicron | 2022-09-20 |
