# Supplementary material for: Genomic Diversity and Evolution of Identified SARS-CoV-2 Variants in Iraq
Source: Pathogens. 2024 Nov 29;13(12):1051. doi: 10.3390/pathogens13121051 (PMC11728743; doi:10.3390/pathogens13121051)
Supplement: Supplementary file 1 [file pathogens-13-01051-s001.zip › pathogens-3308383 supplementary/Table S2.docx]

Table S2. Genetic variations in other structural and non-structural of SARS-CoV-2 in all Iraqi sequences of SARS-CoV-2 as compared to Wuhan-1 strain.

|  | **OMICRON** | **DELTA** | **OTHER** |  |
| --- | --- | --- | --- | --- |
| ORF1a | T3255I (79% of all delta and omicron) | |  |  |
|  | **All omicron**  Deletion of S3675- G3676 (100%)  P3395H (99%)  L3674 (81%).  S135R, T842I, G1307S, L3027F, and T3090 (100% except for 21K)    **21K**  K856R, L2084I, A2710T (100%)  Deletion in S2083- (100%)  I3758V (98%)  V1887I (100% of BA.1.17)  **Other clades**  L3201F (30% of 21L)  (100% of 22D, 23D, 22F , and 23B).  K47R (100% of 23D, 22F, and 23B)  S1221L, P1640S, and N4060S (100% of 22D)  G1819S, and T4175S (100% OF 23D)  L3829F (100% of 23B)  P926H (83% of 23B) | **21J**  T3255I and T3646A (100%)  A1306S, P2046L, P2287S, &V2930L (>98%)  H417R and D1603A (100% of AY.130)  K261N (97% of AY.122 )  T945N (25% of AY.33)  E444A (10% of B.1.617.2)    **21I**  P1640L, A3209V, and V3718A (100%)    T3750I in (92%), A2142V (43%),  Q991H (85%)  deletion of G82- H83- V84- M85- V86- (75% of AY.65) | **20C**  T265I and T346I  -100%    **20B**  T1001I 25%  V2943I 33% of    **Alpha**  T1001I (96%)  L730F(40%), A1708D (96%), I2230T (95%), G3676S (13%), F3677L (14%),  Deletion of S3675- G3676- F3677- (24%) |  |
| ORF 1b | P314L substitution was detected in 92% of all sequences | | | |
|  | **All omicron**  P314L and I1566V ( 99%)  R1315C and T2163I (100% except 21K)  **Other clades**  G662S and S959P (100% of 23D, 22F 23B)  T1050N (83% of 22B)  D1746Y (100% of 23B)  V1092F (79% of 23D) | **All delta**  P314L, and G662S (100%)  P1000L ( 99%)  A1918V (97%)  **21J clade**  I1257V and V1278F (100% of AY.103)  K2557R (100% of AY.126)  V463I (64% of AY.121),  D2333Y (30% of AY.33),  K94R (67% of B.1.617.2)  H1087Y (56% of B.1.617.2)  **21I clade**  M187I (100% of AY.65 ) | **Alpha**  E1871G 19%,  D2333G in 12%,  K1383R in 10% |  |
| Nucleoprotein (N) | R203K/M 94% of all sequences, R203K and G204R in all (omicron, alpha and 20B) sequences | | |  |
|  | P13L ( 98%)  E31- R32- S33- deletion (99%)  S413R (98% of sequences of all clades except 21K clade) | 203M (100%)  D63G ( 97%)  D377Y ( 99%)  G215C(100% of 21J) | **20I Alpha**  D3L in 99% and S235F in 96%  **20A**  M234I (100%),  S194L (100% of B.1.36 & B.1.36.1) D401Y (100% of B.1.438.1 and B.1.438 )  **Other**  T205I (100% 20c)  A220V (100% 20E |  |
| Matrix (M) | Q19E (96 %)  A63T (98% )  D3N (100% of 22B )  D3G instead of N (88% of 21k ) | I82T (99%) |  |  |
| Envelope (E) | T9I (100%)  T11A (100% of 22D,22F, 23A, 23B, 23D) |  |  |  |
| ORF-3a | T223I (100% of all clades except 21K)  W128L(100% of XBB.1.16.2 linage, clade 23B) | S26L (100% of delta seq | 100C (10% of alpha sequences) |  |
| ORF-6 | D61L (100% of all omicron sequences , except clades 21K and 22B). |  |  |  |
| OFR-7a |  | T120I (100%)  W82A (96%) | **Alpha**  Premature termination due to stop codon mutation substitution at Q62* in 34% |  |
| OFR-7b |  | T40I (94%) |  |  |
| ORF-8 | G8* premature termination (81%) in Clades 22F,23B,23D | deletion of D119- F120- (100%) | **Alpha**  R52I, Y73C and Q27* premature termination (>95%) |  |
| ORF-9 | P10S (99%)  deletion in E27- N28- A29- (10%)  D16G (100% of 22B clade)  I5T (94% of 23B and 23D clades) | T60A (100%) |  |  |
