# Supplementary material for: Genomic Diversity and Evolution of Identified SARS-CoV-2 Variants in Iraq
Source: Pathogens. 2024 Nov 29;13(12):1051. doi: 10.3390/pathogens13121051 (PMC11728743; doi:10.3390/pathogens13121051)
Supplement: Supplementary file 1 [file pathogens-13-01051-s001.zip › pathogens-3308383-supplementary/pathogens-3308383 supplementary/Figure S1.pdf]

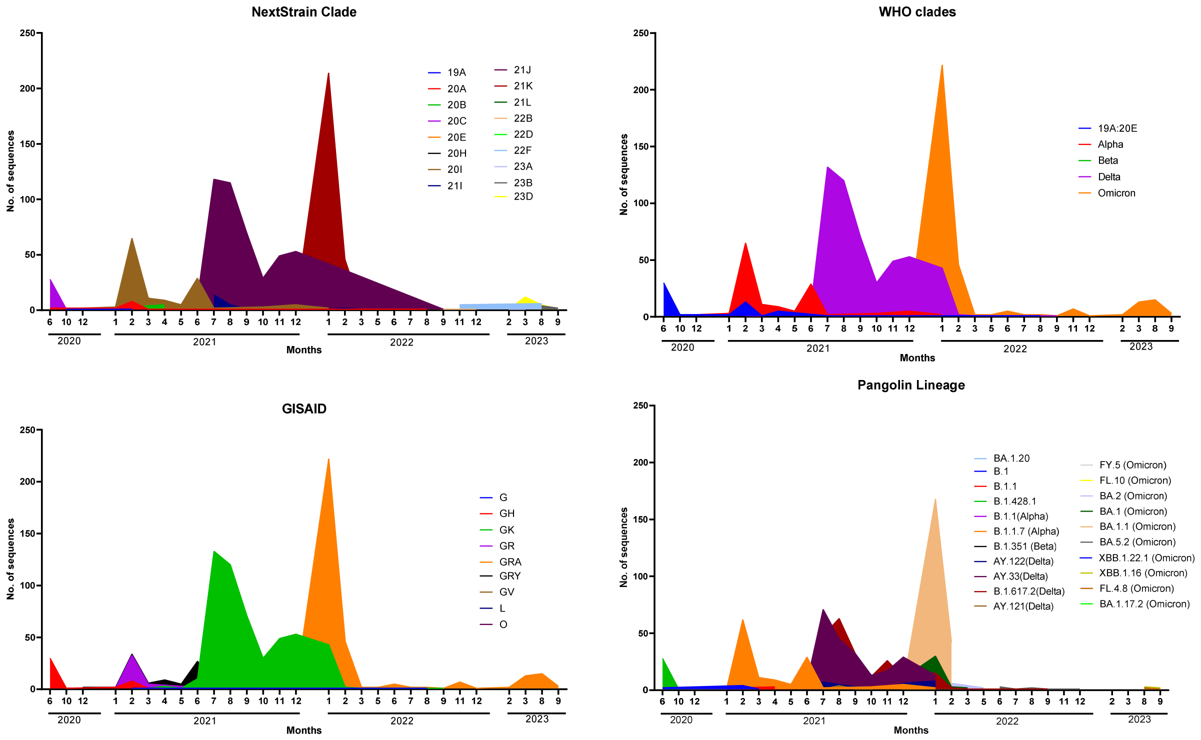


**Figure S1.** Distribution of SARS-CoV-2 variants in Iraq during six pandemic waves assigned using the GISAID clades, Next clade system, and PANGO system.
