# Supplementary material for: Genomic Diversity and Evolution of Identified SARS-CoV-2 Variants in Iraq
Source: Pathogens. 2024 Nov 29;13(12):1051. doi: 10.3390/pathogens13121051 (PMC11728743; doi:10.3390/pathogens13121051)
Supplement: Supplementary file 1 [file pathogens-13-01051-s001.zip › pathogens-3308383-supplementary/pathogens-3308383 supplementary/Table S1.pdf]

Table S1. The accession numbers of the viral genome, collection date, Pangoline lineage, World Health Organization (WHO) name, and Nextstrain clade of sequenced samples in this study.

Virus name	Accession numbers	Pangoline lineage	Nextstrain clade	WHO name	Collection date
hCoV-19/Iraq/NRC-T037N/2023	EPI_ISL_18858302	FL.10	23D	Omicron	2023-02-20
hCoV-19/Iraq/NRC-T038N/2023	EPI_ISL_18861671	XBB.1.5	23A	Omicron	2023-03-12
hCoV-19/Iraq/NRC-T040N/2023	EPI_ISL_18858303	FL.5	23D	Omicron	2023-03-13
hCoV-19/Iraq/NRC-T043N/2023	EPI_ISL_18858304	FL.10	23D	Omicron	2023-03-14
hCoV-19/Iraq/NRC-T046N/2023	EPI_ISL_18858305	XBB.1.9.1	23A	Omicron	2023-03-16
hCoV-19/Iraq/NRC-T047N/2023	EPI_ISL_18858306	XBB.1.5.4	23D	Omicron	2023-03-16
hCoV-19/Iraq/NRC-T052N/2023	EPI_ISL_18861672	FL.2	23D	Omicron	2023-03-18
hCoV-19/Iraq/NRC-T057N/2023	EPI_ISL_18858307	FL.10	23D	Omicron	2023-03-19
hCoV-19/Iraq/NRC-T058N/2023	EPI_ISL_18858308	FL.10	23D	Omicron	2023-03-20
hCoV-19/Iraq/NRC-T060N/2023	EPI_ISL_18861673	FL.4	23D	Omicron	2023-03-20
hCoV-19/Iraq/NRC-T063N/2023	EPI_ISL_18858309	FL.10	23D	Omicron	2023-03-21
hCoV-19/Iraq/NRC-T068N/2023	EPI_ISL_18858310	FL.10	23D	Omicron	2023-03-22
hCoV-19/Iraq/NRC-T076N/2023	EPI_ISL_18858311	FL.4	23D	Omicron	2023-03-23
hCoV-19/Iraq/NRC-T080N/2023	EPI_ISL_18858312	FL.10	23D	Omicron	2023-03-23
hCoV-19/Iraq/NRC-T089N/2023	EPI_ISL_18861674	FL.10	23D	Omicron	2023-03-25
hCoV-19/Iraq/NRC-T001N/2022	EPI_ISL_18861675	BA.5.2	22B	Omicron	2022-08-02
hCoV-19/Iraq/NRC-T020N/2022	EPI_ISL_18858313	BA.5.2.56	22B	Omicron	2022-09-04
hCoV-19/Iraq/NRC-T025N/2022	EPI_ISL_18858314	BA.5.2	22B	Omicron	2022-09-19
hCoV-19/Iraq/NRC-T036N/2022	EPI_ISL_18858315	BA.5.2	22B	Omicron	2022-09-20
