# Supplementary material for: Genomic Diversity and Evolution of Identified SARS-CoV-2 Variants in Iraq
Source: Pathogens. 2024 Nov 29;13(12):1051. doi: 10.3390/pathogens13121051 (PMC11728743; doi:10.3390/pathogens13121051)
Supplement: Supplementary file 1 [file pathogens-13-01051-s001.zip › pathogens-3308383-supplementary/pathogens-3308383 supplementary/Table S2.pdf]

**Table S2.** Genetic variations in other structural and non-structural of SARS-CoV-2 in all Iraqi sequences of SARS-CoV-2 as compared to Wuhan-1 strain.

| | OMICRON | DELTA | OTHER |
|---|---|---|---|
| ORF1a | T3255I (79% of all delta and omicron) | | |
| | **All omicron**<br>Deletion of S3675- G3676 (100%)<br>P3395H (99%)<br>L3674 (81%).<br>S135R, T842I, G1307S, L3027F, and T3090 (100% except for 21K)<br><br>**21K**<br>K856R, L2084I, A2710T (100%)<br>Deletion in S2083- (100%)<br>I3758V (98%)<br>V1887I (100% of BA.1.17)<br>**Other clades**<br>L3201F (30% of 21L)<br>(100% of 22D, 23D, 22F , and 23B).<br>K47R (100% of 23D, 22F, and 23B)<br>S1221L, P1640S, and N4060S (100% of 22D)<br>G1819S, and T4175S (100% OF 23D)<br>L3829F (100% of 23B)<br>P926H (83% of 23B) | **21J**<br>T3255I and T3646A (100%)<br>A1306S, P2046L, P2287S, &V2930L (>98%)<br>H417R and D1603A (100% of AY.130)<br>K261N (97% of AY.122 )<br>T945N (25% of AY.33)<br>E444A (10% of B.1.617.2)<br><br>**21I**<br>P1640L, A3209V, and V3718A (100%)<br><br>T3750I in (92%), A2142V (43%),<br>Q991H (85%)<br>deletion of G82- H83- V84- M85- V86- (75% of AY.65) | **20C**<br>T265I and T346I<br>-100%<br><br>**20B**<br>T1001I 25%<br>V2943I 33% of<br><br>**Alpha**<br>T1001I (96%)<br>L730F(40%), A1708D (96%), I2230T (95%), G3676S (13%), F3677L (14%),<br>Deletion of S3675- G3676- F3677- (24%) |
| ORF 1b | P314L substitution was detected in 92% of all sequences | | |
| | **All omicron**<br>P314L and I1566V ( 99%)<br>R1315C and T2163I (100% except 21K)<br>**Other clades**<br>G662S and S959P (100% of 23D, 22F 23B)<br>T1050N (83% of 22B)<br>D1746Y (100% of 23B)<br>V1092F (79% of 23D) | **All delta**<br>P314L, and G662S (100%)<br>P1000L ( 99%)<br>A1918V (97%)<br>**21J clade**<br>I1257V and V1278F (100% of AY.103)<br>K2557R (100% of AY.126)<br>V463I (64% of AY.121),<br>D2333Y (30% of AY.33),<br>K94R (67% of B.1.617.2)<br>H1087Y (56% of B.1.617.2)<br>**21I clade**<br>M187I (100% of AY.65 ) | **Alpha**<br>E1871G 19%,<br>D2333G in 12%,<br>K1383R in 10% |
| Nucleoprotein (N) | R203K/M 94% of all sequences, R203K and G204R in all (omicron, alpha and 20B) sequences | | |
| | P13L ( 98%)<br>E31- R32- S33- deletion (99%)<br>S413R (98% of sequences of all clades except 21K clade) | 203M (100%)<br>D63G ( 97%)<br>D377Y ( 99%)<br>G215C(100% of 21J) | **20I Alpha**<br>D3L in 99% and S235F in 96%<br>**20A**<br>M234I (100%),<br>S194L (100% of B.1.36 & B.1.36.1) D401Y (100% of B.1.438.1 and B.1.438 ) |

| | | | |
|---|---|---|---|
| | | | **Other**<br>T205I (100% 20c)<br>A220V (100% 20E |
| Matrix (M) | Q19E (96 %)<br>A63T (98% )<br>D3N (100% of 22B )<br>D3G instead of N (88% of 21k ) | I82T (99%) | |
| Envelope (E) | T9I (100%)<br>T11A (100% of 22D,22F, 23A, 23B, 23D) | | |
| ORF-3a | T223I (100% of all clades except 21K)<br>W128L(100% of XBB.1.16.2 linage, clade 23B) | S26L (100% of delta seq | 100C (10% of alpha sequences) |
| ORF-6 | D61L (100% of all omicron sequences , except clades 21K and 22B). | | |
| OFR-7a | | T120I (100%)<br>W82A (96%) | **Alpha**<br>Premature termination due to stop codon mutation substitution at Q62* in 34% |
| OFR-7b | | T40I (94%) | |
| ORF-8 | G8* premature termination (81%) in Clades 22F,23B,23D | deletion of D119- F120- (100%) | **Alpha**<br>R52I, Y73C and Q27* premature termination (>95%) |
| ORF-9 | P10S (99%)<br>deletion in E27- N28- A29- (10%)<br>D16G (100% of 22B clade)<br>I5T (94% of 23B and 23D clades) | T60A (100%) | |
